# The Efficacy of Probiotics in Treating Upper Respiratory Tract Infections, Allergic Rhinitis, and Chronic Rhinosinusitis: A Systematic Review and Meta-Analysis

**DOI:** 10.3390/microorganisms14050986

**Published:** 2026-04-28

**Authors:** Arezki Azzi, Assaf S. Alotaibi, Muath S. Alamri, Mohammed A. Al-Dosari, Faris M. Al Murdhi, Mohammed N. Alatyani, Saad M. Alnojaim, Mohammed A. Alrufayyiq, Mohammed O. Altowaijri

**Affiliations:** 1 Department of Biochemistry, College of Medicine, Imam Mohammad Ibn Saud Islamic University (IMSIU), Riyadh 13317, Saudi Arabia; 2 Health Sciences Research Center (HSRC), Deanship of Scientific Research, Imam Mohammad Ibn Saud Islamic University (IMSIU), Riyadh 13317, Saudi Arabia; 3 College of Medicine, Imam Mohammad Ibn Saud Islamic University (IMSIU), Riyadh 13317, Saudi Arabia; 444004567@sm.imamu.edu.sa (A.S.A.); 444000972@sm.imamu.edu.sa (M.S.A.); 444001398@sm.imamu.edu.sa (M.A.A.-D.); 444003105@sm.imamu.edu.sa (F.M.A.M.); 444004751@sm.imamu.edu.sa (M.N.A.); 444002731@sm.imamu.edu.sa (S.M.A.); 444001139@sm.imamu.edu.sa (M.A.A.); 444004561@sm.imamu.edu.sa (M.O.A.)

**Keywords:** probiotics, upper respiratory tract infections, allergic rhinitis, chronic rhinosinusitis, randomized controlled trial, gut–lung axis

## Abstract

**Background:** Upper respiratory tract infections (URTIs), allergic rhinitis (AR), and chronic rhinosinusitis (CRS) are prevalent and burdensome inflammatory disorders. Probiotics may modulate immune responses via gut–respiratory axis signaling, but their clinical efficacy across these conditions remains uncertain and highly heterogeneous. **Methods:** We conducted a PRISMA-guided systematic review and random-effects meta-analysis of randomized controlled trials (RCTs) evaluating oral or topical probiotics for URTIs, AR, or CRS (MEDLINE, EMBASE, CENTRAL, and Web of Science; inception to July 2025). Disease severity category (acute, subacute, chronic), episode incidence, and duration of illness were extracted alongside symptom scores. Risk of bias was assessed using the Cochrane RoB 2 tool, and certainty of evidence was graded using the GRADE framework. **Results:** Thirty-two RCTs were included. In URTIs, certain strains [e.g., *Lactiplantibacillus plantarum* DR7, *Lactobacillus rhamnosus* GG] reduced symptom duration and antibiotic use; however, the pooled incidence reduction was non-significant (RD = −0.07; 95% CI: −0.23 to 0.09; *p* = 0.38), with very high heterogeneity (I^2^ = 93.12%), limiting interpretability. In AR, probiotics reduced TNSS and improved quality of life (SMDs −0.72 to −2.30) in individual trials supported by immune marker changes [e.g., increased IL-10, decreased IgE]. In CRS, only two small trials—differing in delivery route (topical vs. oral), CRS phenotype, and publication era (2009 and 2017)—with conflicting effect directions were identified; formal meta-analysis was not performed given insufficient and methodologically heterogeneous data, and CRS findings are reported descriptively only. GRADE certainty ranged from very low (URTI incidence) to low (AR symptoms, URTI illness burden). **Conclusions:** Probiotic effects appear strain- and condition-specific. URTI pooled incidence data are unreliable due to extreme heterogeneity; individual strains show consistent benefits on illness burden and AR symptoms/quality of life. Evidence for CRS is insufficient for meta-analytic conclusions; findings are reported descriptively pending adequately powered dedicated trials. Strain-targeted RCTs with standardized outcomes, formal GRADE appraisal, and adequate power are needed before clinical recommendations can be made.

## 1. Introduction

Upper respiratory tract infections [URTIs], including the common cold, pharyngitis, and laryngitis, are one of the most frequent reasons for primary care consultations affecting daily life and performance globally [1,2]. The economic burden is considerable, determined by healthcare costs and lost productivity, with millions of episodes occurring annually in all age groups [3]. Similarly, allergic rhinitis [AR] is a widespread immunoglobulin E [IgE]-mediated inflammatory disorder of the nasal mucosa triggered by exposure to allergens such as pollen, dust mites, and animal dander. It affects an estimated 10–30% of the adult population worldwide and is a major cause of morbidity, significantly impairing the quality of life, sleep, and cognitive function, while also presenting a considerable economic burden through direct medical costs and indirect costs from reduced performance [4,5]. Chronic rhinosinusitis [CRS] is defined as inflammation of the paranasal sinuses continuing for more than 12 weeks [6,7], and the reported prevalence of CRS varies internationally, and may be increasing over time [8]. It is featured by symptoms such as nasal obstruction, rhinorrhea, facial pain, and reduced sense of smell, leading to a severe negative impact on patients’ quality of life and significant healthcare expenditures for medical management [6] and surgical interventions [9].

The conventional management of these conditions often presents challenges. Treatment for URTIs is primarily symptomatic, including analgesics, decongestants, and antipyretics, with antibiotics being inappropriately prescribed despite their ineffectiveness against viral pathogens, thus contributing to the critical issue of antimicrobial resistance [10]. The cornerstone of AR treatment involves allergen avoidance, oral antihistamines, intranasal corticosteroids, and leukotriene receptor antagonists. While effective for many, these treatments can have variable efficacy and side effects [e.g., drowsiness from older antihistamines, nasal irritation from sprays] and do not modify the underlying immune dysregulation [11]. CRS management is multifaceted, typically involving prolonged courses of intranasal corticosteroids, saline irrigations, and occasionally antibiotics or oral corticosteroids for exacerbations; however, the treatment success is often incomplete, and patients may require repeated courses of medication [6] or endoscopic sinus surgery [9].

The role of the human microbiome in immune homeostasis and disease pathogenesis has increasingly become a focal point of research. This has led to rising attention to probiotics, defined as “live microorganisms that, when administered in adequate amounts, confer a health benefit on the host” [12]. Probiotics, principally strains of *Lactobacillus* and *Bifidobacterium*, are most renowned for their gastrointestinal benefits. However, their systemic immunomodulatory effects, mediated in part through the “gut–lung axis” or “gut–respiratory axis,” suggest a potential role in managing respiratory and allergic conditions [13]. This bidirectional communication pathway involves gut microbial metabolites—notably short-chain fatty acids (SCFAs) such as butyrate, propionate, and acetate—that enter systemic circulation and modulate pulmonary and mucosal immune responses. Gut-educated regulatory immune cells can also traffic to distant mucosal sites, including the nasal and bronchial epithelium, via shared mucosal immunity circuits; intestinal dysbiosis has been associated with heightened systemic inflammation and impaired epithelial barrier integrity, both of which may exacerbate allergic and infectious respiratory disease [13,14]. The mechanistic pathways through which probiotics exert their effects include competitive exclusion of pathogens, reinforcement of the epithelial barrier, and, most critically, modulation of the host immune response—encompassing promotion of anti-inflammatory cytokine profiles [e.g., increased interleukin-10 (IL-10) and transforming growth factor-β (TGF-β)], suppression of pro-inflammatory mediators, and rebalancing of T-helper cell responses [e.g., promoting T-regulatory (Treg) cell activity and shifting the Th1/Th2 balance toward a Th1-dominant, anti-allergic phenotype] [14].

Consequently, the potential efficacy of probiotic supplementation has been widely explored as a novel therapeutic or adjunctive strategy for URTIs, AR, and CRS. Probiotics are most commonly administered orally (as capsules, sachets, or fermented dairy products), which represents the most widely studied and clinically practical route of delivery. Intranasal or topical administration directly to the sinonasal mucosa has also been investigated, particularly for AR and CRS, and may confer locally targeted microbiome modulation; however, evidence for this route remains considerably less robust than for oral delivery. Typical therapeutic doses in clinical trials range from 10^8^ to 10^11^ colony-forming units (CFU) per day, with intervention durations of 4 weeks to 6 months [13,14]. This review aims to determine whether probiotics can reduce the incidence and duration of URTIs, alleviate symptom severity and medication reliance in AR, and improve clinical outcomes in CRS. Given the limited and conflicting evidence in CRS (only two eligible trials), findings for that condition are reported descriptively rather than as part of the formal meta-analysis [15,16].

## 2. Materials and Methods

### 2.1. Study Registration and Protocol

This systematic review and meta-analysis were conducted in accordance with the Preferred Reporting Items for Systematic Reviews and Meta-Analyses [PRISMA] guidelines. The protocol was prospectively registered in the PROSPERO database under the registration date of 5 July 2025 with registration number CRD420251086942. The objective was to assess the efficacy of probiotic interventions in three upper airway inflammatory conditions: upper respiratory tract infections [URTIs], allergic rhinitis [AR], and chronic rhinosinusitis [CRS].

### 2.2. Setting and Participants

The included studies were conducted across a wide range of settings and populations, including both pediatric and adult groups. The geographic representation included Europe [e.g., Italy, Sweden, Germany, Switzerland, France], Asia [e.g., Japan, South Korea, Taiwan, China, Malaysia, Vietnam], and the Middle East [e.g., Iran, Pakistan]. The sample sizes varied significantly, from small studies to large multicenter trials.

### 2.3. Eligibility Criteria

Studies were eligible for inclusion if they were designed as randomized controlled trials [RCTs] involving children or adults diagnosed with upper respiratory tract infections [URTIs], allergic rhinitis [AR], or chronic rhinosinusitis [CRS]. The intervention had to involve the administration of probiotics, either orally or topically, delivered through various forms such as capsules, sachets, fermented dairy products, or nasal sprays. Eligible studies were required to include a comparison group receiving either a placebo or standard therapy, such as antihistamines or antibiotics. Furthermore, studies had to report at least one measurable clinical or immunological outcome, including but not limited to symptom scores, duration of infection, or relevant cytokine levels. Exclusion criteria were non-randomized designs, animal/in vitro studies, non-clinical settings, studies without extractable data, or those published only as abstracts or conference proceedings.

### 2.4. Search Strategy

A comprehensive literature search was conducted across four major electronic databases—MEDLINE [via PubMed], EMBASE, the Cochrane Central Register of Controlled Trials [CENTRAL], and Web of Science—from database inception up to July 2025. The search strategy utilized a combination of Medical Subject Headings [MeSH] and free-text keywords to maximize the sensitivity and specificity. Key search terms included “Probiotic” OR “Lactobacillus” OR “Bifidobacterium” AND “Upper respiratory tract infection” OR “URTI” OR “Common cold” OR “Chronic rhinosinusitis” OR “Allergic rhinitis” OR “Hay fever” AND “Treatment” OR “Symptom relief” OR “Outcome” OR “Therapeutic use.” No language restrictions were applied during the initial search. Non-English articles were included if a suitable translation could be obtained. In addition to the database search, the reference lists of all included studies were manually reviewed to identify any additional relevant trials.

### 2.5. Study Selection and Data Extraction

Two independent reviewers screened all titles and abstracts, followed by a full-text review to confirm eligibility. Disagreements were resolved by discussion or judgment by a third reviewer. A standardized data extraction sheet was used to capture the following information:

Study characteristics [e.g., authors, year, country, setting];

Participant demographics [e.g., age, gender, comorbidities];

Intervention details [e.g., probiotic strain, CFU dose, route, duration];

Comparator details;

Primary and secondary outcome measures;

Statistical data [means, SDs, event rates];

Disease severity category (acute, subacute, or chronic), episode incidence, and duration of illness, adverse events, funding sources, and conflicts of interest.

**Figure 1:**
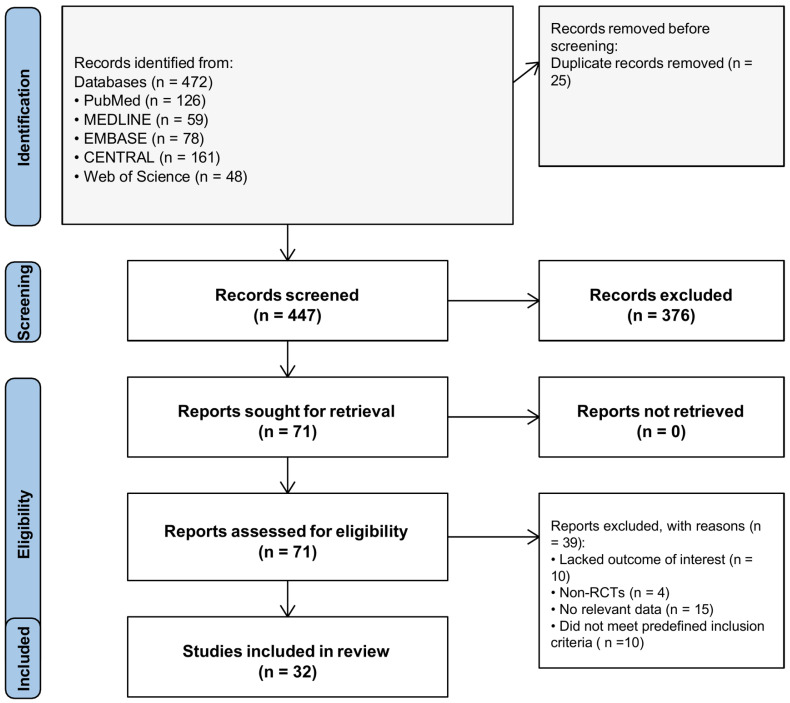
Study selection flow diagram (PRISMA 2020).

The study selection and screening process is summarized in the PRISMA flow diagram (Figure 1).

### 2.6. Outcomes

The primary outcomes were improvements in clinical symptoms for URTIs, AR, and CRS, measured by validated scoring systems [Total Nasal Symptom Score [TNSS], Sino-Nasal-Outcome Test [SNOT-20/22], Rhinoconjonctivitis Quality of Life Questionnaire [RQLQ]. For URTIs, the duration and frequency of infection episodes, antibiotic usage, and healthcare contact were evaluated. For AR, the quality of life and nasal symptom scores were assessed. For CRS, validated sinusitis scores [e.g., SNOT-22] were used.

Secondary outcomes included changes in immunological biomarkers [e.g., IL-6, IFN-γ, IgE, IL-10], adverse events, and other relevant clinical endpoints such as fever duration and medication usage.

### 2.7. Data Synthesis and Statistical Analysis

For meta-analysis, we used the random-effects model to account for between-study heterogeneity. Categorical data were synthesized using risk difference [RD] or risk ratios [RR] with 95% confidence intervals [CI], while continuous outcomes were pooled using standardized mean differences [SMD] or mean differences [MD], depending on the outcome metrics. Heterogeneity was quantified using I^2^ statistics, with I^2^ > 50% indicating moderate-to-high heterogeneity. Tau^2^ and Chi^2^ tests were also reported to estimate variance. Forest plots were constructed to visually represent effect sizes and their confidence intervals.

Funnel plots were constructed for the exploratory assessment of publication bias only. Formal Egger’s test was not performed, as fewer than ten studies were available per analysis, which renders funnel plots unreliable. No definitive conclusions about publication bias should be drawn from these plots.

### 2.8. Subgroup and Sensitivity Analyses

Pre-specified subgroup analyses were conducted by disease condition [URTIs, AR, CRS]. Descriptive comparisons across probiotic strains were made; however, formal meta-regression by strain was not feasible given the few studies per strain and heterogeneous outcomes. Strain-level observations are therefore exploratory.

### 2.9. Data Analysis

The data analysis was conducted using STATA version 17 (StataCorp, College Station, TX, USA, 2021). Risk differences (RD) were used for binary URTI incidence outcomes; standardized mean differences (SMD) were applied for continuous outcomes when studies used different measurement scales; mean differences (MD) were used when identical scales were employed. All forest plot labels specify the metric used. Pooled estimates were calculated with the random-effects model. Heterogeneity was assessed using the I^2^ statistic. Publication bias was explored via funnel plot inspection (exploratory only; formal asymmetry testing not applicable with fewer than 10 studies). Certainty of evidence was assessed using the GRADE approach across five domains: risk of bias, inconsistency, indirectness, imprecision, and publication bias.

## 3. Results

### 3.1. Study Characteristics

A total of 32 randomized controlled trials [RCTs] were identified evaluating the efficacy of probiotics in upper respiratory tract infections [URTIs], allergic rhinitis [AR], and chronic rhinosinusitis [CRS] [17,18,19,20,21,22,23,24,25,26,27,28,29,30,31,32,33,34,35,36,37,38,39,40,41,42,43,44,45,46,47,48,49,50,51,52,53]. The included studies were predominantly double-blind placebo-controlled trials, representing high-level evidence [Level1].

The number of participants varied, ranging from as few as 14 in highly controlled mechanistic studies to over 1100 in large-scale clinical trials. Studies were conducted across different regions, including Europe [Italy, Sweden, France, Germany, Switzerland, Slovenia], Asia [Taiwan, South Korea, Japan, China, Malaysia, Vietnam], and the Middle East [Iran, Pakistan]. Participants were different ages, from infants and young children [mean ages around 1–5 years] to older adults over 65 years. Several studies focused on pediatric cases with allergic rhinitis or recurrent URTIs, while others included healthy adults, middle-aged office workers, or elderly individuals at risk of prolonged respiratory infections. Most trials reported a balanced gender distribution, though a few pediatric studies had incomplete reporting. Where data were available, male and female participants were generally equally distributed. In the large Italian trial, 453 males and 646 females were included. Smaller pediatric cohorts tended to have slight imbalances in gender, often with more boys than girls. As for comorbidities, the majority of trials excluded participants with significant chronic illnesses. However, certain studies deliberately targeted populations with allergic diseases. For instance, in Korean and Taiwanese studies of children with allergic rhinitis, over 90% reported a family history of allergic conditions, and some included children with concurrent mild asthma. In CRS-focused trials, patients with asthma and aspirin sensitivity were represented, whereas otherwise healthy populations were recruited for URTI prevention studies in adults.

### 3.2. Probiotic Intervention

The included studies showed that the majority of probiotic interventions were administered orally, either as capsules, sachets, or fermented dairy products, indicating the practicality and safety of this route for both adult and pediatric populations [19,41]. Only a limited number of trials have explored alternative administration methods, such as intranasal sprays, particularly in allergic rhinitis; however, the evidence remains sparse and less consistent. Considering specific strains, *Lactobacillus rhamnosus GG*, *Lactobacillus casei Shirota*, and *Bifidobacterium lactis* were the most frequently tested and showed favorable effects in reducing the incidence and duration of URTIs [33]. Multi-strain formulations combining Lactobacillus and Bifidobacterium species generally demonstrated more robust improvements in both clinical outcomes and immunological markers compared with single-strain preparations. For allergic rhinitis, strains such as *Lactobacillus paracasei* and *Lactobacillus acidophilus* were associated with significant reductions in nasal symptom scores and improvements in quality-of-life indices [25]. Dosages varied considerably across trials, typically ranging from 10^8^ to 10^11^ CFU/day, with higher doses not always translating into stronger effects, suggesting that the strain selection may be more critical than the absolute dose. Intervention durations ranged from 4 weeks to 6 months, with longer interventions generally correlating with sustained clinical benefit and reduced recurrence of infections [25].

However, heterogeneity in the treatment length and the lack of long-term follow-up in many studies limited conclusions about the durability of the effect.

### 3.3. Probiotic Efficacy

The included clinical trials showed varied but generally favorable outcomes for probiotics in managing upper respiratory tract infections [URTIs], allergic rhinitis [AR], and chronic rhinosinusitis [CRS], though the efficacy varies by strain, dosage, and administration route.

#### 3.3.1. Upper Respiratory Tract Infections [URTIs]

Table 1 presents the study characteristics and effect estimates for the eight trials included in the URTI quantitative synthesis. The results consistently demonstrate that probiotics did not significantly reduce the overall URTI incidence when pooled, but several strains produced meaningful reductions in illness burden (duration, symptom severity) and immunological modulation (Table 1). The specific findings by study are summarized below.

OMNi-BiOTiC^®^ Active (11 strains; 10^10^ CFU/d) [17] did not significantly reduce URTI incidence in older adults ≥ 65 years (30.6% vs. 36.9%; RD −0.063 [−0.197, 0.071]; *p* = 0.354), but it significantly shortened URTI duration (3.1 vs. 6.0 days; *p* = 0.011) and produced anti-inflammatory immune modulation, including reductions in eosinophil (*p* = 0.002) and basophil counts (*p* = 0.001).

A three-strain probiotic mixture (*Bifidobacterium breve* M-16V, *B. lactis* HN019, *L. rhamnosus* HN001) [20] significantly shortened fever duration in children with fever-associated URTIs (median 3 vs. 5 days; adjusted RR = 0.64 [0.51, 0.80]; *p* < 0.001) and reduced pain/discomfort duration (−0.7 days; *p* = 0.006).

*Lactiplantibacillus plantarum* HEAL9 + *L. paracasei* 8700:2 (Probi Defendum^®^; 10^9^ CFU/d) [18] did not reduce URTI incidence in daycare children aged 1–6 years (77.3% vs. 78.2%; RD −0.009; *p* = 0.877), in an underpowered study (106/320 planned participants enrolled). Despite the non-significant incidence result, nasal congestion/runny nose severity was significantly reduced (*p* < 0.05), medication use was lower (*p* = 0.018), and the third URTI episode was shorter (4.0 vs. 6.6 days; *p* = 0.023).

*L. casei* 431 (≥10^9^ CFU/d in acidified milk; 42 days) [24] did not significantly affect influenza vaccine immune responses (the primary outcome) or healthcare contact rates (22.1% vs. 28.2%; RD −0.061; *p* = 0.317) in 1104 healthy adults. However, the URTI symptom duration was significantly shorter in the probiotic group (6.4 vs. 7.3 days; *p* = 0.006), and the symptom severity score was 12% lower.

*L. casei Shirota* fermented milk (10^11^ CFU/d; 12 weeks) [28] produced the largest and most robust incidence reduction among URTI trials (22.4% vs. 53.2%; RD −0.308 [−0.442, −0.173]; *p* < 0.001), with a URTI-free rate of 0.78 vs. 0.47 (log-rank *p* = 0.0008) in healthy male office workers. The common cold incidence was also significantly lower (18.4% vs. 44.7%; *p* = 0.005), and NK cell activity was maintained, while the salivary cortisol elevation was attenuated.

Nasal-sprayed *B. subtilis* + *B. clausii* spores (LiveSpo Navax^®^; 5 × 10^9^ CFU, 3×/day, 7 days) [34] achieved rapid symptom relief in children with acute rhinosinusitis and otitis media with otorrhea: nasal congestion improved in 68.0% of probiotic-treated children vs. 33.3% of controls by day 3 (absolute RD +34.7 percentage points; *p* = 0.007), and rhinorrhea resolved in 96.8% by day 7. The *Streptococcus pneumoniae* bacterial load was reduced approximately 1200-fold, with concomitant reductions in nasal IL-6 and TNF-α.

A five-strain probiotic blend (*L. acidophilus, L. paracasei, Lc. lactis, B. animalis, B. bifidum*; 5 × 10^9^ CFU/d; 30 days) [40] administered before a marathon race significantly reduced post-race URTI symptom prevalence (71% vs. 100%; RD −0.29 [−0.38, −0.20]; *p* < 0.001) and the total WURSS-21 symptom score by 29%, accompanied by lower monocyte IL-6 immediately post-race and higher IL-10 at 1 h. The small sample size (n = 14) and highly specific athletic context limit the generalizability of these findings.

*Heyndrickxia coagulans* SANK70258 [41] reduced the cumulative URTI symptom days by approximately half (13.1 vs. 26.2 days; MD −13.1 days; *p* < 0.001), with all individual symptom scores (runny nose, nasal congestion, sneezing, sore throat) significantly lower. Immune analyses revealed elevated salivary IgA, increased IFNα gene expression via plasmacytoid dendritic cell activation, and higher intestinal butyrate concentrations, with IL-6 and TNFα production by PBMCs suppressed on influenza virus challenge, indicating a gut–immune axis mechanism.

Bertuccioli et al. [27] and Anaya-Loyola et al. [32] reported only qualitative descriptors (e.g., “significantly reduced”) without numeric data; these studies were excluded from quantitative pooling and are described narratively only. Chong et al. [31] reported a mean difference (0.32 fewer URTI episodes); without control group means or SDs, the SE was not computable, and this study was excluded from quantitative synthesis.

**Figure 2:**
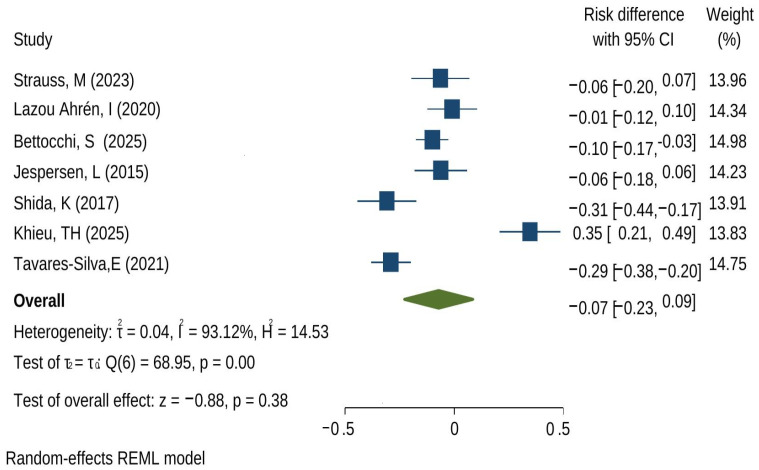
Forest plot for URTI incidence (risk difference) in probiotics vs. control. The green diamond is the pooled prevalence, the blue squares show size effects, and the lines represent confidence intervals. (references Strauss et al. [17], Lazou Ahrén et al. [18], Bettochi et al. [20], Jesperson et al. [24], Shida et al. [28], Khieu et al. [34], Tavares-Silva et al. [40]).

Across seven trials reporting URTI incidence, the random-effects meta-analysis showed no statistically significant overall reduction in URTI risk with probiotics compared with control (risk difference [RD] = −0.07; 95% CI: −0.23 to 0.09; *p* = 0.38) (Figure 2). The confidence interval spans both potential benefit and harm, indicating uncertainty in the average effect across studies.

**Figure 3:**
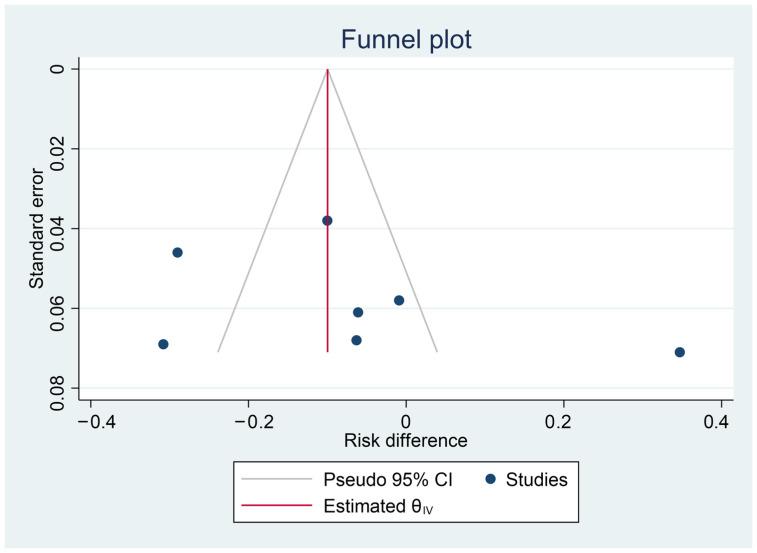
Funnel plot for URTI incidence (risk difference) in probiotics vs. control.

The funnel plot (Figure 3) is presented for exploratory purposes. With only seven studies in this analysis, funnel plots lack statistical power to detect publication bias reliably, and no formal asymmetry test was applied. This visual should not be interpreted as confirming the absence of publication bias.

A key limitation of this synthesis is the very high between-study heterogeneity (I^2^ = 93.12%; τ^2^ = 0.04), suggesting that the effect of probiotics on URTI incidence varies substantially across trials. This inconsistency is evident in the spread of individual effects—from moderate reductions in URTI risk (e.g., Shida et al. [28]; Tavares-Silva et al. [40]) to a positive absolute risk difference in one study reflecting symptom relief rather than incidence (ARD +0.35) [34]. Differences in participant age/risk profile, URTI case definitions, probiotic strain(s) and dose, intervention duration, and follow-up likely contribute to this heterogeneity; therefore, the pooled estimate should be interpreted as an average across non-equivalent interventions rather than a single ‘class effect’.

#### 3.3.2. Allergic Rhinitis [AR]

The efficacy of probiotics in treating allergic rhinitis (AR) is reported in Table 2. Heat-killed *L. paracasei* LCW23 [19] improved nasal symptoms [runny nose *p* = 0.001, congestion *p* = 0.033] and QoL [95% of items improved by week 12]. *B. longum* IM55 + *L. plantarum* IM76 [21] reduced TNSS [*p* = 0.011] and increased IL-10 [*p* = 0.047], indicating immune modulation. Synbiotics [23] lowered IgE [*p* = 0.035] and improved miniRQLQ scores [*p* = 0.04] as adjuncts to standard therapy. Enterococcus faecalis [30] reduced TNSS [*p* = 0.00, CSMS [*p* = 0.009], though the QoL changes were nonsignificant.

**Figure 4:**
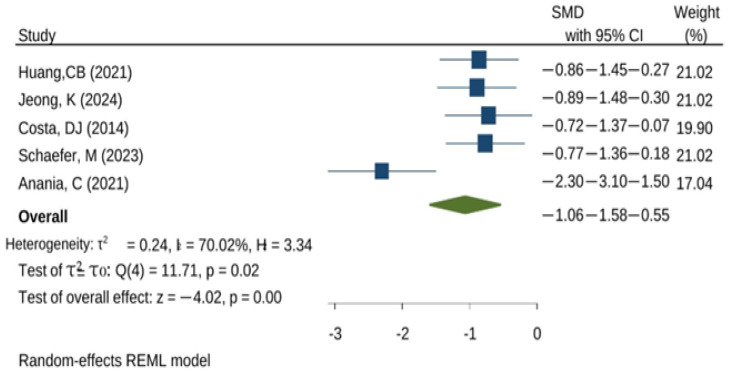
Forest plot for allergic rhinitis outcomes (standardized mean difference) in probiotics vs. control. The green diamond is the pooled prevalence, the blue squares show size effects, and the lines represent confidence intervals. (references Huang et al. [19], Jeong et al. [21], Costa et al. [29], Schaefer et al. [30], Anania et al. [25]).

Figure 4 summarizes the quantitative synthesis of allergic rhinitis outcomes. Most trials favored probiotics (negative standardized mean differences indicating symptom reduction), and several confidence intervals did not cross the line of no effect, supporting a statistically significant benefit in those studies. The between-study variability in effect size and precision likely reflects differences in populations, probiotic strains/formulations, duration of supplementation, and the symptom/QoL instruments used.

#### 3.3.3. Neutral/Negative Findings

Nasal-sprayed *L. rhamnosus* SP1, *L. paracasei* 101/37, and *Lactococcus lactis* L1A [39] produced no significant improvement in TNSS, Mini-RQLQ, Peak Nasal Inspiratory Flow, or Fractional Exhaled Nitric Oxide” (FeNO). Although a minor innate immune response was observed within the probiotic treatment period compared with the baseline, no cytokine or mediator change was found versus the placebo, with the sole exception of a minor increase in IL-17/IL-17A within the probiotic run. No TNF-α change versus the placebo was reported. These results indicate that nasal delivery of this specific probiotic assemblage does not alleviate allergic rhinitis symptoms under nasal allergen challenge conditions.

*L. paracasei* LP-33 [29] showed a mixed response in allergic rhinitis: the RQLQ global score improved significantly versus the placebo (*p* = 0.0255), and ocular symptom scores also improved (*p* = 0.0029), but the rhinitis total nasal symptom score did not reach significance (*p* = 0.1288). This pattern of QoL and ocular benefit without nasal symptom reduction is noted here as a partial clinically nuanced finding warranting cautious interpretation.

#### 3.3.4. Chronic Rhinosinusitis [CRS]—Narrative Description Only

Given that only two trials met the inclusion criteria for CRS—differing substantially in the delivery route, CRS phenotype, and publication era (see Table 3)—formal meta-analysis was not performed; the results are presented descriptively only. Topical lactic acid bacteria microbiome [46] and oral probiotics [47] both failed to demonstrate a statistically significant between-group improvement in SNOT-20/22 scores, and the effect directions conflicted across the two studies (SMD −0.51 vs. +0.32), underscoring the insufficient and inconsistent evidence for CRS.

### 3.4. Risk of Bias Assessment

Table 4 presents Cochrane RoB 2 domain-level assessments for the 16 included trials. Among the eight URTI trials, three were rated Low overall risk of bias—Bettocchi [20], Wada [26], and Aida [41]—while five received Some Concerns: Strauss [17], Lazou Ahrén [18], and Jespersen [24] due to incomplete outcome-selection reporting (Domain 5), and Shida [28] and Bertuccioli [27] due to additional concerns in missing-data handling (Domain 3) and, for Bertuccioli, allocation concealment (Domain 1). No URTI trial was rated at High overall risk. Among the six AR trials, four were rated Low overall (Huang [19], Jeong [21], Anania [25], Schaefer [30]); Faridzadeh [23] and Costa [29] received Some Concerns, principally in Domains 3 and 5. For CRS, Mårtensson [46] received Some Concerns (Domains 3 and 5), while Mukerji [47] was the sole trial rated High overall risk of bias—reflecting concerns across randomization (D1), missing data (D3), and outcome-selection reporting (D5), compounded by an extremely small sample (n = 14). Overall, the evidence base is predominantly of acceptable methodological quality; however, residual uncertainty in selective reporting and allocation concealment warrants cautious interpretation of the pooled estimates.

#### Certainty of Evidence (GRADE Summary)

The certainty of evidence across four formally meta-analyzed outcomes, assessed using the GRADE framework, ranged from Low to Very Low (see Table S1, Supplementary Material). The URTI incidence reduction was rated Very Low certainty, downgraded for extreme heterogeneity (I^2^ = 93.12%), serious risk of bias, and imprecision. The AR outcomes—both TNSS reduction and quality of life (RQLQ)—were rated Low certainty, with effect sizes that approach but do not definitively exceed accepted minimal clinically important differences. The URTI illness burden received Low certainty, supported by consistent directional benefit across multiple individual trials. The CRS findings (Table 3) are reported as a descriptive narrative only and have been excluded from the formal GRADE assessment, given that only two small trials with conflicting effect directions and substantial methodological heterogeneity were available; no pooled estimate or formal certainty rating is therefore assigned for CRS. These ratings underscore the preliminary nature of the current evidence and highlight the need for larger standardized trials before firm clinical recommendations can be issued.

## 4. Discussion

The present analysis of 32 randomized controlled trials [RCTs] provides a nuanced and stratified understanding of the role of probiotic interventions in the management of upper respiratory tract infections [URTIs], allergic rhinitis [AR], and chronic rhinosinusitis [CRS]. The findings highlight a central belief of probiotic therapy: efficacy is not a class effect but is deeply strain-specific and condition-dependent and affected by the delivery method and duration. The high level of evidence [Level 1] from predominantly double-blind placebo-controlled trials gives considerable weight to these conclusions, though the significant heterogeneity in the study populations, interventions, and outcomes necessitates a cautious and detailed interpretation.

Clinical trials demonstrate that probiotics exert strain-specific and condition-targeted effects, particularly in URTIs and AR, while showing limited efficacy in CRS. Across the URTI trials, the pooled evidence did not demonstrate a statistically significant reduction in URTI incidence [15,48]. Nevertheless, individual randomized trials—such as those evaluating OMNi-BiOTiC^®^ Active [17] and *Lactiplantibacillus plantarum* DR7 [31]—reported clinically meaningful improvements in secondary outcomes, including shorter symptom duration [34], shorter fever duration in children [20], and reduced overall medication and antibiotic use [17,24].

The significant benefits observed with specific formulations like OMNi-BiOTiC^®^ Active [17], *L. casei Shirota* [28], and *B. subtilis/clausii* [34] demonstrate that only certain strains possess the immunomodulatory and antimicrobial properties sufficient for meaningful URTI benefit, whether for incidence reduction, illness duration, or fever attenuation [20]. These benefits are mechanistically supported by the consistent findings of immune modulation, particularly reductions in pro-inflammatory cytokines like IL-6 and elevations in immunoregulatory markers like IFN-γ [40]. Taken together, these findings suggest that probiotics may be more effective at attenuating the illness burden than at preventing URTI occurrence outright. The interpretation is limited by the substantial heterogeneity across trials, which likely reflects differences in populations and baseline risk, URTI definitions and ascertainment, and—most importantly—variation in probiotic strains, formulations, dosing, and duration of use. Accordingly, pooled estimates should be viewed as an average across non-equivalent interventions, and strain-specific conclusions remain difficult without more standardized outcomes and pre-specified subgroup analyses.

Notably, intranasal delivery may represent a distinct mucosa-directed approach; for example, the intranasal application of Bacillus spores in pediatric acute respiratory conditions has been associated with symptomatic benefits in some settings [34]. However, confirmatory trials in clearly defined URTI phenotypes are required to establish the generalizability, optimal dosing, and safety.

Compared with URTIs, the allergic rhinitis trials showed a more consistently favorable direction of effect on symptom scores and quality-of-life measures. Across studies, probiotics were associated with improvements in patient-reported outcomes, supporting their potential role as adjuncts to standard AR management in selected patients. Several trials also reported concordant immunologic changes—such as increases in IL-10 and/or reductions in IgE [23] or Th2 [21]-skewed markers—providing biologic plausibility for clinical benefit through the modulation of allergic inflammation [29,30].

Across AR trials, probiotics showed directionally consistent improvements in symptom scores and quality of life, supported by concordant immunological changes (e.g., increased IL-10, reduced IgE, Th1/Th2 rebalancing) [36,38]. The improvement in quality-of-life scores reported by Huang et al. [19] represents a meaningful patient-centered endpoint. However, clinical relevance requires careful contextualization: the minimal clinically important difference (MCID) for TNSS is approximately 1.0 point, and for RQLQ, it is approximately 0.5 points. Because heterogeneous measurement instruments were used across studies, direct back-translation of the reported SMDs (−0.72 to −2.30) into scale units was not possible; it is therefore recommended to avoid equating statistical significance with clinical meaningfulness without further scale-specific validation. Another study [29] found an improvement in the global RQLQ but not in TNSS, suggesting that subjective well-being and objective nasal symptoms may not correlate perfectly and that different strains affect these domains differently. Furthermore, the neutral result from the intranasal spray of *L. rhamnosus* SP1 [39], despite changes in immune markers [increased TNF-α], reinforces that not all administration routes or strains are effective, even within the same pathology underscoring the likelihood of strain-, route-, and phenotype-specific effects. [39]. This body of evidence strongly supports the use of specific probiotic strains as a valuable adjunct to standard AR therapy, potentially reducing the reliance on antihistamines and nasal corticosteroids. Similarly, a randomized controlled trial showed that combining probiotics with budesonide [a corticosteroid] led to significantly better quality of life and symptom relief than budesonide alone [37]. Moreover, a systematic review and meta-analysis reported notable improvements in quality-of-life scores and nasal symptoms in patients receiving probiotics, further supporting their beneficial role when used alongside conventional treatments [49,50,51,52,53].

In contrast to AR, probiotic interventions in chronic rhinosinusitis (CRS) were largely ineffective in the available evidence, with limited trials showing no consistent improvement in validated symptom measures (e.g., SNOT-20/22). Overall, the current literature is insufficient to support routine probiotic use for established CRS. CRS involves multifactorial pathophysiology (biofilms, chronic inflammation, and, in many patients, anatomic or polyp-related contributors) that may limit the ability of standard oral probiotics to meaningfully alter the sinonasal microbiome.

These null findings suggest that CRS may require longer, topical, or otherwise more targeted microbiome-based strategies to achieve measurable clinical effects. Future work may be better directed toward topical or peri-operative approaches and well-characterized formulations, with careful endotyping and standardized endpoints to identify whether any subgroup derives benefit.

## 5. Conclusions

In conclusion, this analysis of 32 RCTs provides a condition-stratified overview of probiotic efficacy, but the results must be interpreted against substantial methodological limitations. Probiotics are not a monolithic therapy; the effects appear strain- and condition-specific. For URTIs, selected strains reduced the illness burden, but the pooled incidence reduction was non-significant (RD −0.07; *p* = 0.38) with very high heterogeneity (I^2^ = 93.12%), precluding a definitive class-level conclusion. For AR, several strains showed improvements in TNSS and quality of life with Low GRADE certainty; clinical magnitudes require validation against established MCIDs before recommendations can be issued. For CRS, only two small trials (differing in delivery route, CRS phenotype, and publication era) with conflicting effect directions were identified; formal meta-analysis was not performed, and the findings are strictly descriptive and hypothesis-generating. Observed strain-level differences are hypothesis-generating and require confirmation via formal meta-regression in adequately powered trials. For clinicians, these findings suggest cautious consideration of specific strains (e.g., *L. rhamnosus* GG, *L. casei Shirota* for URTIs; *L. paracasei*, B. longum for AR) only in conjunction with an understanding of the low certainty of evidence. For the research community, priority areas include standardized outcome reporting, strain-specific RCTs, GRADE-based synthesis, and dedicated CRS trials. Future trials should prioritize clearly defined strains, standardized outcomes, adequate follow-up, and improved reporting to support clinical translation.

## Figures and Tables

**Table 1:** Study characteristics for the efficacy of probiotics in treating upper respiratory tract infections (URTIs).

Study [Ref]	Probiotic Strain/Product	Population	Primary Outcome Reported in Table	Probiotic Group ^§^	Control Group ^§^	Effect Size [95% CI]	*p*-Value	Key Additional Effect (from Source) ^$^
Strauss et al. [17]	OMNi-BiOTiC^®^ Active (11 strains; 10^10^ CFU/d)	Older adults ≥ 65 y (n = 95); 12 weeks	URTI incidence (non-significant)	30.6%	36.9%	RD −0.063 [−0.197, 0.071]	*p* = 0.354	↓ URTI duration: 3.1 vs. 6.0 days (*p* = 0.011); ↓ eosinophils (*p* = 0.002); ↓ basophils (*p* = 0.001)
Lazou Ahrén et al. [18]	*L. plantarum* HEAL9 + *L. paracasei* 8700:2 (10^9^ CFU/d; Probi Defendum^®^)	Children 1–6 y, daycare (n = 106 PP); 3 months	≥1 URTI episode (non-significant; underpowered)	77.3%	78.2%	RD −0.009 [−0.123, 0.105]	*p* = 0.877	↓ Nasal congestion severity (*p* < 0.05); ↓ medication use (*p* = 0.018); ↓ daycare absence; episode 3 duration 4.0 vs. 6.6 days (*p* = 0.023)
Bettocchi et al. [20]	B. breve M-16V + *B. lactis* HN019 + *L. rhamnosus* HN001 (0.5 mL/d; 14 days)	Children 28d–4 y with fever + URTI (n = 128); ED-based RCT	Fever duration (primary outcome; corrected)	Median 3 days [IQR 2–4]	Median 5 days [IQR 4–6]	aRR 0.64 [0.51, 0.80]	*p* < 0.001	↓ Pain/discomfort: −0.7 days (*p* = 0.006); antibiotic prescriptions after discharge: no significant difference
Jespersen et al. [24]	L. casei 431 (≥10^9^ CFU/d in acidified milk; 42 days)	Healthy adults 18–60 y (n = 1104); 2 centers	Healthcare contact rate (non-significant)	22.1%	28.2%	RD −0.061 [−0.179, 0.057]	*p* = 0.317	↓ URTI symptom duration: 6.4 vs. 7.3 days (*p* = 0.006); symptom severity score 12% lower; no effect on influenza vaccine response
Shida et al. [28]	*L. casei Shirota* (LcS; 10^11^ CFU/d fermented milk; 12 weeks)	Healthy male office workers 30–49 y (n = 96)	URTI incidence	22.4%	53.2%	RD −0.308 [−0.442, −0.173]	*p* < 0.001	Common cold incidence 18.4% vs. 44.7% (*p* = 0.005); ↑ NK cell activity maintained; ↓ salivary cortisol rise inhibited; URTI-free rate 0.78 vs. 0.47 (log-rank *p* = 0.0008)
Khieu et al. [34]	*B. subtilis + B. clausii* spores (LiveSpo Navax^®^; 5 × 10^9^ CFU nasal spray; 3×/d, 7 days)	Children 1 mo–12 y with ARS+AOM (n = 61 completers)	Nasal congestion relief by day 3 (proportion improved)	68.0%	33.3%	ARD +34.7 pp [+12.7, +56.7]	*p* = 0.007	Rhinorrhea resolution by day 7: 96.8% vs. ~50% (1.94×); S. pneumoniae load ↓ ~1200-fold; ↓ nasal IL-6 and TNF-α; bacteria migrated to middle ear via Eustachian tube
Tavares-Silva et al. [40]	5-strain blend (*L. acidophilus, L. paracasei, Lc. lactis*, *B. animalis, B. bifidum;* 5 × 10^9^ CFU/d; 30 days)	Male marathon runners (n = 14); post-race assessment	URTI symptom prevalence (WURSS-21) post-race	71%	100%	RD −0.29 [−0.38, −0.20]	*p* < 0.001	↓ IL-6 post-race (*p* < 0.05); ↑ IL-10 at 1 h post (*p* < 0.05); WURSS-21 total score 29% lower. Note: n = 14, highly specific athletic population
Aida et al. [41] †	Heyndrickxia coagulans SANK70258 (spore-forming LAB; 12 weeks)	Japanese adults (n not pooled); winter season	Cumulative URTI symptom days	13.1 days	26.2 days	MD −13.1 days [SE N/A]	*p* < 0.001 †	↑ Salivary IgA; ↑ IFNα gene expression (pDC activation); NK cell activity tendency ↑; ↑ intestinal butyrate; ↓ IL-6 and TNFα on influenza virus challenge

$ The arrow indicate the direction of the changes either ↑ increase or ↓decrease. † Aida et al. [41]: group means published directly; SE not calculable from available data; *p* < 0.001 stated by authors; ARD = absolute risk difference; aRR = adjusted risk ratio; MD = mean difference; pp = percentage points; ARS = acute rhinosinusitis; AOM = acute otitis media; pDC = plasmacytoid dendritic cell; sIgA = secretory IgA. § Values in the “Probiotic Group” and “Control Group” columns represent (a) event rates expressed as percentages (proportion of per-protocol participants experiencing ≥1 URTI episode) for studies reporting incidence outcomes or (b) group means (±SD where available) for studies reporting continuous endpoints (e.g., days of illness, symptom scores). Percentages are calculated as the number of participants with at least one qualifying event divided by the per-protocol group total. Where a range is given (e.g., median [IQR]), the study used a non-parametric distribution; refer to the primary source for full distributional data.

**Table 2:** Study characteristics for the efficacy of probiotics in treating allergic rhinitis (AR).

Study	Outcome Metric	Probiotic Mean/Median [SD/IQR]	Placebo Mean/Median [SD/IQR]	SMD [95% CI]	SE	*p*-Value
Huang et al. (2021) [19]	Nasal congestion score	1.2 [0.8]	2.1 (1.2)	−0.86 [−1.45, −0.27]	0.3	0.033
Jeong et al. (2024) [21]	TNSS (evening)	4.2 [1.5]	5.8 (2.0)	−0.89 [−1.48, −0.30]	0.3	0.004
Costa et al. (2014) [29]	RQLQ global score	2.1 [1.0]	2.9 (1.2)	−0.72 [−1.37, −0.07]	0.33	0.0255
Schaefer et al. (2023) [30]	TNSS	5.3 [2.1]	7.1 (2.5)	−0.77 [−1.35, −0.19]	0.3	0.001
Anania (2021) [25]	Nasal Symptom Score [NSS]	5.4 [3.2]	13.6 (4.1)	−2.30 [−3.10, −1.50]	0.41	<0.001

**Table 3:** Study characteristics for the efficacy of probiotics in treating chronic rhinosinusitis (CRS).

Study	Outcome Metric	Probiotic Mean/Median [SD/IQR]	Placebo Mean/Median [SD/IQR]	SMD [95% CI]	SE	*p*-Value
Mårtensson et al. [46]	SNOT-22 score	38.0 [12.5]	34.0 [11.8]	0.32 [−0.29, 0.93]	0.31	0.082
Mukerji et al. [47]	SNOT-20 score [week 4]	1.8 [1.2]	2.5 [1.5]	−0.51 [−0.99, −0.03]	0.25	0.79

Mårtensson [46] reports medians and interquartile ranges (IQR) analyzed with Wilcoxon signed-rank test (crossover design, n = 20); values shown are median [IQR bound]. Mukerji [47]: *p* = 0.79 is the between-group comparison at week 4; the within-group change from baseline in the probiotic arm was significant (*p* = 0.002), but no between-group difference was observed at week 4 (*p* = 0.79) or week 8 (*p* = 0.23). The SMD for Mukerji [47] was derived from endpoint group means and SDs and does not reflect the between-group test result; it is presented for pooling purposes only.

**Table 4:** Cochrane RoB 2 risk-of-bias traffic-light summary for included RCTs.

Study [Ref]	D1: Randomization	D2: Deviations	D3: Missing Data	D4: Outcome Meas.	D5: Reporting	Overall RoB	Condition
Strauss 2023 [17]	L	L	L	L	SC	SC	URTI
Lazou Ahrén 2020 [18]	L	L	SC	L	L	SC	URTI
Bettocchi 2025 [20]	L	L	L	L	L	L	URTI
Jespersen 2015 [24]	L	L	L	L	SC	SC	URTI
Shida 2017 [28]	L	L	SC	L	L	SC	URTI
Wada 2024 [26]	L	L	L	L	L	L	URTI
Bertuccioli 2024 [27]	SC	L	SC	L	SC	SC	URTI
Aida 2024 [41]	L	L	L	L	L	L	URTI
Huang 2021 [19]	L	L	L	L	L	L	AR
Jeong 2024 [21]	L	L	L	L	L	L	AR
Faridzadeh 2023 [23]	L	L	SC	L	L	SC	AR
Anania 2021 [25]	L	L	L	L	L	L	AR
Costa 2014 [29]	L	L	SC	L	SC	SC	AR
Schaefer 2023 [30]	L	L	L	L	L	L	AR
Mårtensson 2017 [46]	L	L	SC	L	SC	SC	CRS
Mukerji 2009 [47]	SC	L	SC	L	SC	H	CRS

RoB 2 domains: D1 = randomization process; D2 = deviations from intended interventions; D3 = missing outcome data; D4 = measurement of the outcome; D5 = selection of the reported result. Risk: L = Low (green); SC = Some concerns (yellow); H = High (red). Assessments reflect published trial reporting and were not adjudicated independently by the trial authors.

## Data Availability

The original contributions presented in the study are included in the article/Supplementary Material, further inquiries can be directed to the corresponding author.

## Supplementary Materials

The following supporting information can be downloaded at https://www.mdpi.com/article/10.3390/microorganisms14050986/s1. PRISMA 2020 checklist. Table S1. GRADE Summary of Findings: Probiotic interventions in URTIs, AR, and CRS. Full domain-level GRADE ratings with effect estimates and certainty symbols for five primary outcomes.

## Abbreviations

The following abbreviations are used in this manuscript:

URTIs	Upper respiratory tract infections
AR	Allergic rhinitis
GRADE	Grades of recommendation, assessment, development, and evaluation
CRS	Chronic rhinosinusitis
RCTs	Randomized controlled trials
IL-10	Interleukin-10
IL-6	Interleukin-6
IgE	Immunoglobulin E
IFN-γ	Interferon-gamma
TNF-α	Tumor necrosis factor-alpha
Th1	T helper type 1
Th2	T helper type 2
CFU	Colony-forming unit
TNSS	Total Nasal Symptom Score
NSS	Nasal Symptom Score
RQLQ	Rhino conjunctivitis Quality of Life Questionnaire 1
SNOT	Sino-Nasal Outcome Test
QoL	Quality of life
MeSH	Medical Subject Headings
CSMS	Combined Symptom–Medication Score.
LAB	Lactic acid bacteria
*L. rhamnosus* GG	*Lactobacillus rhamnosus* GG
*L. rhamnosus* SP1	*Lactobacillus rhamnosus* SP1
*L. casei* Shirota	*Lactobacillus casei* Shirota
*B. lactis*	*Bifidobacterium lactis*
*L. paracasei*	*Lactobacillus paracasei*
*L. plantarum*	*Lactiplantibacillus plantarum* (formerly *Lactobacillus plantarum*; reclassified 2020)
