# Supplementary material for: The Efficacy of Probiotics in Treating Upper Respiratory Tract Infections, Allergic Rhinitis, and Chronic Rhinosinusitis: A Systematic Review and Meta-Analysis
Source: Microorganisms. 2026 Apr 28;14(5):986. doi: 10.3390/microorganisms14050986 (PMC13209766; doi:10.3390/microorganisms14050986)
Supplement: Supplementary file 1 [file microorganisms-14-00986-s001.zip › microorganisms-4232316-supplementary.pdf]

Microorganisms — Supplementary Material

The Efficacy of Probiotics in Treating Upper Respiratory Tract Infections, Allergic Rhinitis, and Chronic Rhinosinusitis: A Systematic Review and Meta-Analysis

Table S1. GRADE Summary of Findings: Probiotic Interventions in URTIs and AR (formally meta-analyzed); CRS included for contextual reference only (†).

Outcome	Studies, N (Participants)	Risk of Bias	Inconsistency	Indirectness	Imprecision	Effect Estimate (Random-Effects)	GRADE Certainty
**URTI — Incidence (RD)**	7 RCTs (n ≈ 1,820)	Serious ↑ a	Very serious ↑↑ b	Serious ↑ c	Serious ↑ d	RD −0.07 (95% CI −0.23 to 0.09); $p = 0.38$	⊕○○○ Very Low
**URTI — Illness duration/burden**	~10 RCTs (n ≈ 2,100)	Some concerns e	Moderate ↑ f	Serious ↑ c	Not serious	MD −1.8 to −13.1 days (varies by study); several $p < 0.05$	⊕⊕○○ Low
**AR — TNSS / Nasal Symptom Score**	5 RCTs (n ≈ 540)	Some concerns e	Moderate ↑ g	Not serious	Not serious	SMD −0.77 to −2.30; multiple $p < 0.05$; MCID for TNSS ≈ 1.0 pt	⊕⊕○○ Low
**AR — Quality of Life (RQLQ)**	3 RCTs (n ≈ 320)	Some concerns e	Not serious	Not serious	Serious ↑ h	SMD −0.72 (95% CI −1.37 to −0.07); MCID for RQLQ ≈ 0.5 pt	⊕⊕○○ Low
**CRS — SNOT-20/22** *† Narrative only — not meta-analyzed (see footnote ℓ)*	2 RCTs (n ≈ 34)	Some concerns e	Serious ↑ i	Serious ↑ j	Very serious ↑↑ k	SMD −0.51 to +0.32 (conflicting directions across 2 trials); no pooled estimate calculated; contextual reference only	⊕○○○ Very Low * ** Contextual only*

Abbreviations: RD, risk difference; SMD, standardized mean difference; MD, mean difference; TNSS, Total Nasal Symptom Score; RQLQ, Rhinoconjunctivitis Quality of Life Questionnaire; SNOT, Sino-Nasal Outcome Test; MCID, minimal clinically important difference; AR, allergic rhinitis; CRS, chronic rhinosinusitis; URTI, upper respiratory tract infection. **Certainty symbols:** ⊕ = domain met (not downgraded); ○ = downgraded. * = contextual reference only (not formally meta-analyzed).

Footnotes:

[a] Unclear allocation concealment in 2/7 trials.

[b] $I^2 = 93.12\%$; Chi^2 $p < 0.001$; extreme between-study heterogeneity.

[c] Mixed adult/paediatric populations; variable URTI and AR definitions across trials.

[d] 95% CI spans benefit and null; point estimate non-significant.

[e] Unclear blinding of outcome assessors in one or more trials.

[f] I^2 45–70% across URTI illness duration outcomes; moderate heterogeneity.

[g] Different symptom scales used (TNSS, Nasal Symptom Score, nasal congestion score).

[h] Only 3 trials with RQLQ data; varying follow-up durations.

[i] Conflicting direction of effect between the two CRS trials (SMD −0.51 vs. +0.32).

[j] Heterogeneous CRS phenotypes (CRSsNP vs. unspecified) and delivery routes (topical vs. oral).

[k] Very small total sample (n ≈ 34 across 2 trials); very serious imprecision.

[ℓ] CRS row is presented for contextual reference only and is NOT part of the formal meta-analysis. Only two trials were eligible (Mårtensson et al., 2017 [topical]; Mukerji et al., 2009 [oral]); they differ substantially in delivery route, CRS phenotype (CRSsNP vs. unspecified), and publication era, and their effect directions conflict (SMD −0.51 vs. +0.32). Formal pooling was therefore not performed. GRADE domain ratings are provided descriptively to characterize the evidence base but should not be interpreted as a formal certainty assessment. Footnotes i, j, and k apply exclusively to this descriptive row.
